# gCoSRNA: Generalizable Coaxial Stacking Prediction for RNA Junctions Using Secondary Structure

**DOI:** 10.3390/biom16020230

**Published:** 2026-02-02

**Authors:** Shasha Li, Qianqian Xu, Ya-Lan Tan, Jian Jiang, Bengong Zhang, Ya-Zhou Shi

**Affiliations:** 1 Center for Applied Mathematics and Interdisciplinary Sciences, School of Mathematics & Statistics, Wuhan Textile University, Wuhan 430200, China; 2 School of Bioengineering and Health, Wuhan Textile University, Wuhan 430200, China

**Keywords:** RNA 3D structure, coaxial stacking, RNA junctions, machine learning

## Abstract

Coaxial stacking between adjacent stems is a key tertiary interaction that defines the spatial organization of RNA junctions, which are core structural motifs in folded RNAs. The accurate prediction of coaxial stacking is critical for RNA 3D structure modeling, yet existing computational tools remain limited, especially for junctions with variable numbers of branches or complex topologies. Here, we present gCoSRNA, a generalizable computational framework for predicting coaxial stacking configurations using RNA sequence and secondary structure as input. Instead of developing separate models for each junction type, gCoSRNA decomposes multi-way junctions into all possible adjacent stem pairs, termed pseudo two-way junctions, and uses a unified RF classifier to evaluate stacking probabilities. Global stacking configurations are inferred by integrating these pairwise predictions, eliminating the need for explicit junction type classification. Benchmarking on two independent test sets (297 RNA junctions), including CASP15/16 and RNA-Puzzles targets, shows that gCoSRNA achieves consistently high accuracy (mean ~0.89) across junctions with two to seven branches, outperforming existing junction-specific methods. These results highlight the model’s ability to capture higher-order structural features and its potential utility in RNA tertiary structure prediction pipelines.

## 1. Introduction

RNA plays a central role in a wide range of biological processes, including gene regulation, catalysis, and molecular recognition, which critically depend on RNA’s ability to fold into complex three-dimensional structures [1,2]. Multi-way junctions, where three or more helices are connected through loop regions, are widespread in structured RNAs and play essential roles in their function [3,4,5]. For instance, hammerhead ribozymes adopt defined coaxial stacking patterns that bring helices into close proximity, creating compact active sites for catalytic self-cleavage [3]. Riboswitches rely on similar multibranched folds to recognize small molecules and regulate downstream gene expression [4]. Transfer RNAs (tRNAs), with their conserved cloverleaf secondary structure, fold into an inverted L-shaped three-dimensional conformation that enables the accurate delivery of amino acids during translation [5]. Understanding RNA structure therefore not only is essential for uncovering its biological functions but also provides a foundation for RNA structure-based drug design and RNA nanotechnology [6,7].

Due to the high cost and limited scalability of experimental RNA structure determination (e.g., X-ray diffraction, nuclear magnetic resonance, and cryo-electron microscopy) [8,9,10], some computational methods have been developed for predicting RNA 3D structures [11,12,13,14,15,16]. These approaches generally fall into three main categories: physics-based modeling [17,18,19,20,21,22,23,24,25,26,27,28], fragment assembly [29,30,31,32,33,34], and AI-based methods [13,35,36,37,38,39,40]. Although AI-based RNA structure prediction methods have advanced rapidly following the success of AlphaFold2 [40,41], recent results from CASP15 and CASP16 suggest that these approaches have not yet outperformed traditional physics-based and fragment assembly methods [42,43]. CASP16 further emphasized that RNA 3D structure prediction remains largely template-dependent [43]. However, the number of experimentally determined RNA structures, particularly those containing multi-way junctions, remains limited, constraining the accuracy of fragment-based methods for complex RNAs [14,15]. While coaxial stacking between stems connected through junctions is a key interaction shaping RNA topology, accurately modeling this interaction from physics remains a significant challenge [44,45,46]. This highlights the need for reliable computational tools specifically designed to predict coaxial stacking in RNA junctions, which could be further integrated into physics-based frameworks to improve the overall accuracy of RNA 3D structure prediction [8,9].

To classify RNA three-way junctions, Lescoute and Westhof et al. proposed an algorithm that groups junctions into three families based on sequence and base-pairing patterns, derived from 32 known RNA structures [47]. Similarly, Laing and Schlick analyzed 62 RNAs with four-way junctions and identified nine structural families, outlining the key features of each [48]. Junctions within the same family share a common coaxial stacking arrangement, allowing the coaxial stacking of unknown RNAs to be inferred through family-level classification [49,50]. However, the predictive power of these methods is limited by the small number of available training structures and the relatively coarse nature of family-level classification [12,13,14]. To directly predict coaxial stacking from sequence and secondary structure, Laing et al. developed Junction Explorer, which combines a set of secondary structure features with a random forest (RF) classifier to predict coaxial stacking in three- or four-way junctions [49]. However, this method requires separate models for different junction types, making it highly dependent on the availability of structurally resolved homologs [49].

In this study, we present gCoSRNA, a generalizable machine learning framework for predicting coaxial stacking in RNA junctions based solely on secondary structure and sequence information. Unlike previous approaches that rely on junction-specific models or family-level classifications, we introduced a new conceptual strategy: any multi-way junction can be decomposed into all possible adjacent stem–stem pairs (pseudo two-way junctions, e.g., H1–H2, H2–H3, and H3–H1 in a three-way junction with three stems H1, H2, and H3). A single unified model is trained to estimate stacking probabilities for these stem pairs, and the global coaxial stacking configuration is then reconstructed from the pairwise predictions. This pairwise decomposition strategy effectively expands the training data and allows for robust generalization across diverse junction types and topologies. By exploiting widely available secondary structure-derived features, gCoSRNA provides not only a practical predictor but also a framework for understanding the modular principles that govern RNA junction architecture.

## 2. Materials and Methods

### 2.1. Pairwise Decomposition of Multi-Way Junctions

In contrast to RNA Junction-Explorer, which treats the entire secondary structure of each RNA junction as a single training sample [49], gCoSRNA decomposes every *n*-way junction into multiple pseudo two-way junctions, each serving as an independent sample. Specifically, a pseudo two-way junction is defined as a pair of adjacent stems along with all intervening unpaired nucleotides (e.g., loop regions) that connect them. As illustrated in Figure S1, a three-way junction with stems H1, H2, and H3 is decomposed into three pseudo two-way junctions: H1–H2, H2–H3, and H3–H1. Each pseudo two-way junction is assigned a binary label indicating whether its two stems are coaxially stacked in the native 3D structure.

### 2.2. Feature Extraction from Pseudo Two-Way Junctions

For each pseudo two-way junction (comprising stems H1 and H2), we extracted 12 features derived from sequence and secondary structure information [49]:

(1) *L*0: Length of the loop directly connecting H1 and H2. For true two-way junctions (i.e., stems separated by an internal loop or bulge), *L*0 refers to the length of the 5′-loop connecting H1 and H2.
(2) *L*1 and *L*2: Number of unpaired nucleotides at the terminal ends of H1 and H2, respectively. If H1 and H2 are directly connected, L1 = L2, both corresponding to the 3′-side loop length between the two stems.
(3) *A*(*L*0), *A*(*L*1), and *A*(*L*2): Number of consecutive adenines within loops *L*0, *L*1, and *L*2, respectively [51].
(4) *L*(*H*1) and *L*(*H*2): Lengths of stems H1 and H2, measured by the number of base pairs.
(5) *N*: Number of intervening stems located between H1 and H2 along the junction loop in the original *n*-way junction (Figure S2). For *n* ≥ 3, one side of the stem pair may be interrupted by additional stems, and *N* reflects this relative spatial separation.

(6) ${\Delta G}_{37}^{{}^{\circ}}(H1)$ and ${\Delta G}_{37}^{{}^{\circ}}(H2)$*:* Free energy (${\Delta G}_{37}^{{}^{\circ}}$) of stems H1 and H2, respectively.

(7) ${\Delta G}_{37}^{{}^{\circ}}(H1-H2)$: Coaxial stacking free energy between H1 and H2, estimated from base stacking interactions at their junction interface [52,53,54,55].

To characterize the thermodynamic stability of each pseudo two-way junction, we computed three types of free energy features (${\Delta G}_{37}^{{}^{\circ}}(H1)$, ${\Delta G}_{37}^{{}^{\circ}}(H2)$, and ${\Delta G}_{37}^{{}^{\circ}}(H1-H2)$ based on the RNA nearest-neighbor thermodynamic model developed by Turner’s group [53,54]. The free energy of each helix, ${\Delta G}_{37}^{{}^{\circ}}(H1)$ and ${\Delta G}_{37}^{{}^{\circ}}(H2)$, was calculated using the Watson–Crick–Franklin (WCF) model, as a linear combination of initiation energy, AU-end penalties, symmetry correction, and base stacking contributions [53]:

(1) $$\Delta G_{37,WCF}^{{}^{\circ}}=\Delta G_{37,init}^{{}^{\circ}}+\Delta G_{37,AU-end}^{{}^{\circ}}+\Delta G_{37,sym}^{{}^{\circ}}+\Delta G_{37,stacking}^{{}^{\circ}}$$

The coaxial stacking free energy between two adjacent helices, ${\Delta G}_{37}^{{}^{\circ}}(H1-H2)$, was estimated using the Turner nearest-neighbor parameters according to the following empirical function [52]:

(2) $${\Delta G}_{37}^{{}^{\circ}}(H1-H2)=\{\begin{array}{c} {∆G}_{37,stack}^{\left(0\right)},\;{(L}_{0}=0)\\ {∆G}_{37,stack}^{\left(1\right)}+\Delta G_{37,mismatch}^{{}^{\circ}},\;{(L}_{0}=1)\\ a+b\cdot L_{0}+2c,\;{(2\leq L}_{0}\leq 6)\\ a\;+\;6b\;+\;1.1\;*\;ln\left(\frac{L_{0}}{6}\right)+\;2c,\;{(L}_{0}>6) \end{array}\;$$

Here, ${∆G}_{37,stack}^{\left(0\right)}$ and ${∆G}_{37,stack}^{\left(1\right)}$ are coaxial stacking free energies with 0 and 1 unpaired nucleotide (i.e., *L*0) inserted between the two helices, respectively [53]. The term $\Delta G_{37,mismatch}^{{}^{\circ}}$ in Equation (2) represents the terminal mismatch penalty (2.1 kcal/mol). The empirical constants of *a* = 9.3, *b* = 0, and *c* = −0.6 were derived from Refs. [52,56] and Turner’s 2004 parameters (https://rna.urmc.rochester.edu/NNDB/turner04/index.html, accessed on 1 December 2023). Previous studies have shown that the likelihood of coaxial stacking decreases sharply with increasing loop length between stems [47,55]. In particular, when *L*0 ≥ 2, stacking becomes thermodynamically less favorable. Accordingly, a penalty term is applied as formulated in Equation (2) to reflect this reduced stacking propensity for longer loops.

### 2.3. Training and Test Datasets

As shown in Figures S3 and S4, a total of 2534 RNA structures were retrieved from the Protein Data Bank (PDB, as of September 2024). To perform an initial coarse reduction in global redundancy, CD-HIT(version 4.8.1) [57] was applied to whole chains with an 80% sequence identity threshold, yielding 452 representative RNA structures (Figure S4). We noted that this global threshold cannot fully exclude homologous RNAs, particularly within large ribosomal complexes (e.g., *Thermus thermophilus* structures) where evolutionary conservation maintains structural similarity despite sequence divergence. Given the limited availability of experimentally determined RNA 3D structures and our specific focus on junction-level rather than whole-molecule prediction, such homologous RNAs were retained at this stage.

Since complex RNAs (e.g., ribosomal RNAs, ribonucleases, and group II introns) generally contain multiple junctions, each retained PDB structure was analyzed using DSSR (version 1.8.1) (Dissecting the Spatial Structure of RNA) [58] and systematically decomposed into its constituent *n*-way junctions, ranging from two-way to seven-way junctions (Figure S5). Here, a junction is defined as the central junction loop along with all directly connected stems, while other loop types (e.g., hairpin loops) and tertiary interactions between different junctions were intentionally excluded for simplicity. This definition may neglect certain loop–loop interactions or ligand-binding effects that could modulate coaxial stacking. For each extracted junction, the sequence, dot–bracket secondary structure, PDB identifier, chain identifier, and nucleotide ranges were explicitly recorded. To rigorously minimize data leakage, redundancy was further reduced at the junction level using a multi-stage filtering strategy inspired by RNA3DB [59,60,61]. First, junction sequences were clustered using CD-HIT(version 4.8.1) with a 90% identity threshold to remove nearly identical sequence variants. Second, the remaining junctions were pairwise-aligned at the secondary structure level using the Needleman–Wunsch algorithm on dot–bracket representations (scoring: +2 for matches, −1 for mismatches, −2 for gaps). Junctions sharing greater than 90% secondary structure similarity were considered redundant, and only one representative was retained. Finally, for junctions with available three-dimensional coordinates, pairwise structural similarity was evaluated using the TM-score [62]. Junction pairs with an RMSD < 2.0 Å and TM-score > 0.7 were classified as structurally redundant, and a single representative was kept.

Although residual homology cannot be entirely eliminated, this multi-level filtering ensured that only junctions with distinct secondary and tertiary structures were retained. The final dataset comprised 1157 junctions, including 960 two-way, 87 three-way, 91 four-way, 15 five-way, 1 six-way, and 3 seven-way junctions. For model development, junctions within each *n*-way category were randomly split, with approximately 85% assigned to the training set and the remaining 15% forming Test Set I. However, due to the scarcity of six- and seven-way junctions, all such instances were exclusively allocated to Test Set I to rigorously evaluate the generalizability of gCoSRNA to complex, unseen topologies.

To further assess model generalizability, an independent Test Set II was constructed from RNA-Puzzles and CASP15/16 targets [12,42,43]. Junctions in this set were filtered to remove any sequence, secondary structure, or tertiary structure redundancy with the training data using the same criteria described above. The resulting Test Set II contains 77 two-way, 9 three-way, and 28 four-way junctions (Figure S6) and serves as a strictly independent benchmark.

Finally, all junctions in the training and test sets were decomposed into pseudo two-way junctions and processed through a unified feature extraction pipeline for model training and evaluation.

### 2.4. Model Training and Inference Strategy

Using the constructed dataset, we trained a binary RF classifier to predict whether two stems within a pseudo two-way junction are coaxially stacked. Given the high-dimensional and limited-sample nature of RNA coaxial stacking data, we adopted a Bayesian optimization strategy to tune model hyperparameters efficiently. Six key hyperparameters were optimized: the number of trees, maximum tree depth, minimum samples required to split an internal node, minimum samples at a leaf node, feature sampling ratio, and the class weight assigned to positive samples. A Gaussian process model, coupled with 10-fold cross-validation, was employed to explore the hyperparameter space. The optimization procedure began with five random initializations, followed by ten iterations of guided search. The final optimized configuration was as follows: 703 trees, a maximum depth of 43, minimum split size of 5, minimum leaf size of 1, feature sampling ratio of 0.23, and a positive class weight of 4.8.

During inference, each *n*-way junction is decomposed into all cyclically adjacent pseudo two-way junctions. For each pair, the trained RF model outputs a binary stacking label together with a probability p ∈ [0, 1]. To reconstruct the coaxial stacking configuration of the entire junction from these pairwise predictions, we formulated the task as a maximum weight matching problem on a cycle graph. Specifically, we represented the junction as a cycle graph *G* = (*V*,*E*), where the vertices correspond to stems *V* = {s_1_, …, s_n_}, and the edges represent adjacency relations E = {(s_i_,s_i+1_): i = 1, …, n}. Each edge (s_i_,s_i+1_) is weighted by its predicted probability *p_i_*, and only edges with *p_i_* > 0.42 are retained in the graph. This threshold was determined based on the intersection point of the predicted probability distributions for positive and negative samples estimated using kernel density estimation (KDE) on the training set (Figure S7). A matching M ⊆ E is defined as a subset of edges such that no two edges share a vertex. The optimal stacking configuration is obtained by solving the following:

(3) $$M^{*}\;=\;\underset{M\subseteq \mathrm{E matching}}{\arg\max}\sum_{(u,v)\in M}p_{uv}.$$

Intuitively, this approach identifies the set of non-overlapping stem–stem stacks that collectively maximize the total predicted probability, yielding a coherent junction-level configuration.

### 2.5. Re-Implementation of RNA Junction-Explorer for Benchmarking

RNA Junction-Explorer is an RF-based method originally developed to predict coaxial stacking configurations in RNA junctions using features derived from the secondary structure [49]. It remains the only publicly reported tool specifically designed for this purpose. The method extracts junction-level features (e.g., 15, 18, and 10 features for three-, four-, and high-order junctions), such as loop length, nucleotide composition, and helix-end stacking energies, and trains separate classifiers for junctions of different orders (e.g., three-way and four-way) [49]. However, the official web server (https://nature.njit.edu/biosoft/Junction-Explorer/, accessed on 1 November 2023) is no longer functional and fails to return valid results, likely due to discontinued maintenance. To facilitate a direct comparison with our gCoSRNA method, we re-implemented the RNA Junction-Explorer pipeline according to the algorithmic details described in its original publication [49].

We first replicated model training using the original feature sets and training data (e.g., 110 three-way junctions), along with the hyperparameters reported in the original RNA Junction-Explorer publication [49]. To enable a more rigorous comparison, we then retrained the model, referred to as Junction-Explorer-n, using our newly curated and non-redundant RNA junction dataset while keeping the original feature definitions unchanged. For retraining, we applied Bayesian optimization with 10-fold cross-validation to re-tune model hyperparameters for improved generalizability.

### 2.6. Evaluation Metrics

To comprehensively assess the performance of gCoSRNA across different junction orders and classification settings, we employed a combination of standard and task-specific metrics (see Supplementary Materials for details). For the binary classification task of predicting coaxial stacking between two stems in (pseudo) two-way junctions, we reported accuracy, F1-score, and area under the ROC curve (AUC), all computed from the standard confusion matrix. In the multi-class setting for three-way and four-way junctions, where the number of possible stacking topologies increases to four and seven, respectively (Figure S8), we reported the overall classification accuracy and included the full confusion matrices to highlight specific patterns of misclassification.

For junctions with five or more stems, the combinatorial complexity of full-topology classification becomes prohibitive. Following the convention used in RNA Junction-Explorer [49], we instead focused on the accuracy of local pairwise stacking decisions between adjacent stem pairs. Specifically, we defined pairwise accuracy (PA) as follows:

(4) $$PA=\frac{1}{n}\sum_{\left(i,j\right)}1[{pred}_{ij}=y_{ij}],\;$$

where *n* is the number of cyclically adjacent stem pairs (*H_i_*, *H_i+_*_1_) in an *n*-way junction, *y_ij_* denotes the ground truth (stacked/unstacked), and *pred_ij_* is the model prediction (see Figure S9). This metric captures the fidelity of local stacking predictions while remaining tractable for higher-order junctions.

## 3. Results

### 3.1. Overview of gCoSRNA

**Figure 1:**
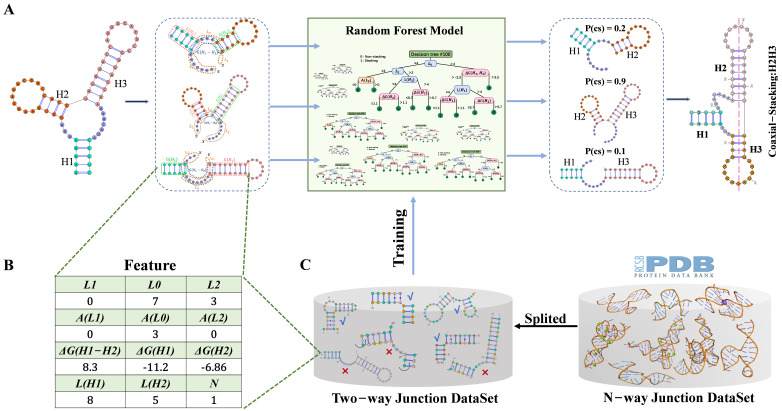
Overview of gCoSRNA framework for coaxial stacking prediction. (**A**) Schematic workflow of gCoSRNA. Representative RNA three-way junction with three helices (H1, H2, and H3) is decomposed into three adjacent stem pairs (H1–H2, H2–H3, and H1–H3) based on secondary structure. Each pair is independently evaluated by pretrained RF model to estimate probability of coaxial stacking. These pairwise probabilities are then integrated to infer overall stacking configuration of junction (e.g., coaxial stacking between H2 and H3). (**B**) Example feature set used for each stem pair, incorporating sequence- and secondary structure-based features. (**C**) Training pipeline of RF model using features derived from experimentally resolved RNA 3D structures.

gCoSRNA is a machine learning-based framework for predicting coaxial stacking patterns in RNA multi-way junctions using secondary structure-derived features. As illustrated in Figure 1A, the framework consists of two main stages: (1) each multi-way junction is decomposed into pseudo two-way junctions formed by adjacent stem pairs (e.g., three pairs for a three-way junction; see Figure S1), and stacking probabilities are predicted for each pair using a pretrained RF model; (2) the pairwise predictions are then integrated to reconstruct the complete coaxial stacking configuration of the junction. The RF model is trained on experimentally resolved RNA 3D structures using features extracted from both the primary sequence and secondary structure (Figure 1B), including loop lengths and thermodynamic stability (Figure 1C). Model parameters are optimized via tenfold cross-validation to ensure robustness and generalizability. This decomposition-based strategy enables gCoSRNA to achieve accurate and scalable predictions across a wide range of RNA junction architectures. Further methodological details are provided in Section 2.

### 3.2. Reproducibility of Junction-Explorer

To validate the reproducibility of RNA Junction-Explorer, we first re-implemented and retrained the model using the original feature sets, dataset, and hyperparameters as described in its seminal publication [49]. When evaluated using 10-fold cross-validation on the original dataset, our implementation achieved prediction accuracies of 81% for three-way junctions and 77% for four-way junctions, which are fully consistent with the reported results [49]. This confirms the correctness and fidelity of our re-implementation.

We further trained an enhanced version, Junction-Explorer-n, using our newly curated and expanded junction dataset. To ensure fair comparison on the original dataset, we conducted 10 rounds of random sub-sampling, each time withholding one-tenth of the original samples as a test set. The average prediction accuracies for three-way and four-way coaxial stacking reached 82% and 79%, respectively, showing improvements of approximately ~1.2% and 2.6% over the original model. These results suggest that training with more comprehensive data can improve Junction-Explorer’s performance, though the gains are relatively modest (i.e., <3%).

### 3.3. gCoSRNA Performance Evaluation

We first assessed the predictive performance of gCoSRNA using 10-fold cross-validation on the training set of pseudo two-way junctions. As shown in Figure S10, the model achieved consistently high accuracy across all folds, with an average area under the ROC curve (AUC) of 0.92 and fold-wise AUCs exceeding 0.90. Other evaluation metrics, including accuracy, precision, recall, F1-score, and Cohen’s Kappa, also demonstrated strong and stable performance, e.g., all folds yielded accuracy and F1-scores above 0.80, with recall consistently maintained above 0.85 and precision near 0.85. These results highlight the strong discriminative power and stability of gCoSRNA in identifying coaxial stacking from pseudo two-way junction data.

Although gCoSRNA was trained exclusively on pseudo two-way junctions, it can be readily applied to RNA junctions of arbitrary order by predicting the coaxial stacking status of each pair of adjacent stems. Two-way junctions have a binary outcome, stacked or unstacked. For three-way junctions, four possible configurations exist: H1–H2, H2–H3, H3–H1, or no stacking (Figure S8). Four-way junctions exhibit seven distinct patterns, including four single-stack cases (H1–H2, H2–H3, H3–H4, and H4–H1), two double-stack cases (H1–H2 and H3–H4 and H2–H3 and H4–H1), and a no-stacking configuration. For these junctions (order < 5), a prediction was considered correct only if the full coaxial stacking pattern matched the known annotation exactly. For junctions with five or more stems, where the number of possible configurations grows rapidly, we focused instead on pairwise accuracy (PA); see Equation (4) in Section 2. This metric assesses whether the stacking prediction for each adjacent stem pair is correct, providing a tractable means of evaluating performance in high-order junctions. We evaluated the predictive performance of gCoSRNA on the two independent test sets (Table 1).

#### 3.3.1. On Test Set I

**Figure 2:**
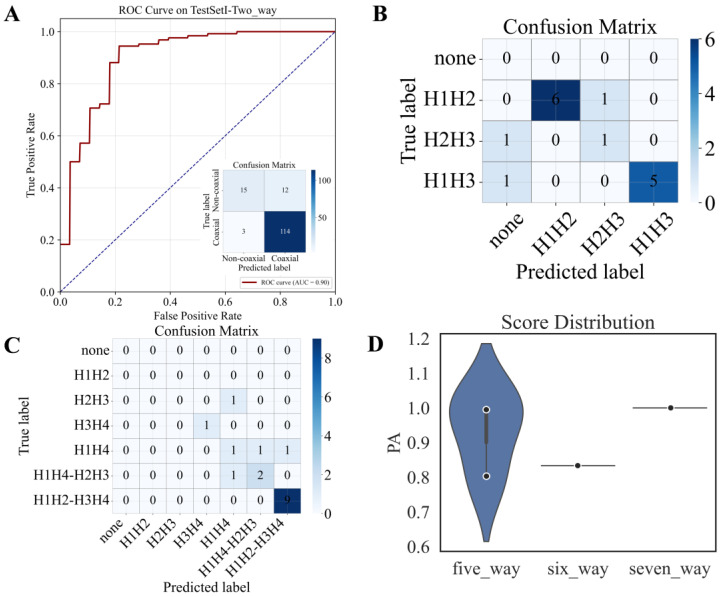
Performance of gCoSRNA on Test Set I. (**A**) ROC curve for coaxial stacking prediction in two-way junctions, with corresponding confusion matrix shown as inset. (**B**,**C**) Confusion matrices for coaxial stacking prediction in three-way (**B**) and four-way (**C**) junctions. (**D**) Distribution of pairwise accuracy (PA) values for five-, six-, and seven-way junctions, visualized as violin plots.

Test Set I comprised a diverse collection of RNA junctions with a distribution similar to the training set (see Section 2.3). As shown in Table 1 and Table S1, gCoSRNA achieved its highest performance on two-way junctions, with an AUC of 0.90 (Figure 2A) and an overall accuracy of 0.90 (Table 1) (F1-score of 0.94; see Table S1), highlighting the consistency between native and pseudo two-way stacking features. The confusion matrix reveals that gCoSRNA correctly predicted 129 out of 144 coaxially stacked stem pairs (Figure 2A). However, only ~55.5% of non-stacked pairs were accurately classified, likely due to the strong class imbalance in the test set, where stacked configurations were heavily overrepresented relative to non-stacked ones (Figure S6B).

For three-way and four-way junctions, gCoSRNA maintained strong performance with accuracies of 0.80 and 0.76, respectively (Table 1). The confusion matrices indicate that most stacking types were well-predicted, though specific configurations (e.g., H2–H3 in three-way and H1H4 in four-way junctions) showed lower sensitivity (Figure 2B,C), suggesting possible local feature ambiguity or limited training representation for these patterns. These results further support that accurate coaxial stacking prediction between adjacent stem pairs is sufficient to reconstruct the overall stacking topology of RNA junctions.

For five-way junctions, gCoSRNA achieved a pairwise accuracy (PA) of 0.93. Notably, despite the complete absence of six- and seven-way junctions in the training set, the model demonstrated robust generalizability to these complex topologies. It achieved an average PA exceeding 0.95 on the six- and seven-way junctions in the test set (*n* = 4), as shown in Table 1 and Figure 2D. Representative examples of five-, six-, and seven-way junctions with distinct coaxial stacking patterns are shown in Figure S11, where gCoSRNA recovered nearly all native coaxial stacking interactions, with only minimal false negatives. These results demonstrate the model’s robust extrapolation capacity to underrepresented and structurally complex junction types, validating its broad applicability to RNA tertiary structure modeling.

#### 3.3.2. On Test Set II

Test Set II was curated from RNA-Puzzles and CASP RNA targets, which are structurally diverse RNAs with no significant sequence overlap with the training set. Among the 77 two-way junctions, gCoSRNA achieved 0.95 accuracy (Table 1) and a 0.97 F1-score (Table S1), indicating strong generalizability to unseen RNA structures. Performance on nine three-way junctions dropped to 0.66 accuracy, likely due to limited sample size and increased structural variability, particularly within RNA–ligand/ion interactions (see Figure S12). In contrast, prediction on 28 four-way junctions remained robust, with 0.75 accuracy (Table 1), underscoring the model’s ability to handle moderate structural complexity. These results highlight both the adaptability and current limitations of gCoSRNA, suggesting that incorporating RNA–protein complex data during training may further improve generalization in future applications [63].

### 3.4. Comparison with Existing Methods

To further evaluate the performance of gCoSRNA, we compared it against two versions of Junction-Explorer. Both models were applied to the three-way and four-way junctions in Test Set I and Test Set II (Table 1). Consistent with the training set results, Junction-Explorer-n achieved better average accuracy (0.74) than the original Junction-Explorer (0.48) for three- and four-way junctions, but both were significantly worse than gCoSRNA (0.78). In Test Set I, gCoSRNA attained accuracies of 0.80 and 0.76 on three-way and four-way junctions, respectively. Compared to Junction-Explorer-n, these represent improvements of 10% and 0%, and relative to Junction-Explorer, the gains were 10% and 204%, respectively. In Test Set II, although gCoSRNA’s accuracy on three-way junctions (0.66) was lower than that in Test Set I (0.80), it still notably outperformed Junction-Explorer (0.55). Specifically, among the nine three-way junctions, gCoSRNA correctly predicted six, while Junction-Explorer correctly predicted five. There were three RNAs for which both methods failed, possibly due to ligands/ion-induced conformational shifts or tertiary constraints not captured by the training data (Figure S12). For the 28 four-way junctions, gCoSRNA and Junction-Explorer-n achieved correct predictions on 25 and 22 cases, respectively. Overall, these comparisons reveal that building junction-specific models (e.g., for three-way or four-way junctions) does not confer a clear advantage over gCoSRNA, which reconstructs the global stacking configuration by independently predicting pairwise stem stackings. This suggests that the abundant and structurally diverse two-way junctions (native or pseudo) can serve as effective training surrogates for learning generalizable coaxial stacking rules.

To further assess the performance of gCoSRNA against physics-based approaches, we compared it with the free energy-based method of Tyagi and Mathews [55]. For consistency, we adopted the same benchmark of 30 RNA junction structures used in their study. After removing sequences with >80% similarity to our training set and excluding several non-standard junctions, the final test set included 17 three-way and 19 four-way junctions. On this benchmark, gCoSRNA correctly predicted 9 of the three-way and 16 of the four-way junctions (Figure S13; Table S2), substantially exceeding the 6 and 9 correct predictions reported by Tyagi and Mathews. These results demonstrate that gCoSRNA surpasses the physics-based model by integrating thermodynamic parameters with data-driven structural learning.

### 3.5. Feature Importance Analysis

To investigate the contribution of individual features to gCoSRNA’s performance, we analyzed feature importance scores derived from the trained RF model using two standard split-based criteria, Gini impurity and information gain; see Figure S14. Both metrics evaluate how effectively a feature partitions the data, with higher scores indicating greater contributions to the model’s decision-making process across the ensemble. The top-ranked features include the intrinsic stability (free energy) of the two stems (${\Delta G}_{37}^{{}^{\circ}}(H1)$ and ${\Delta G}_{37}^{{}^{\circ}}(H2)$), the loop length connecting them (L0), the number of intervening stems (N) between them in the original *n*-way junction (N), and the coaxial stacking free energy between adjacent stems (${\Delta G}_{37}^{{}^{\circ}}(H1-H2)$)). These results are consistent with biophysical principles indicating that coaxial stacking is mainly shaped by thermodynamic preferences and spatial arrangement [49,53].

Statistical analysis indicates that individual stem properties, such as stem size and intrinsic stability, which were not included in Junction-Explorer [49], show significant differences between stacking and non-stacking pseudo two-way junctions (*p* < 0.0001; see Figure S15), even though the distributions appear visually similar. To further evaluate their contribution, we performed ablation experiments. Removing these stem-level features reduced the average tenfold cross-validation accuracy from 0.86 to 0.82, suggesting that stem stability and size, while not independently decisive, provide complementary information that improves the discrimination of stacking versus non-stacking pairs. Similarly, the omission of the topological descriptor *N* led to a broader distribution of cross-validation accuracies and a modest decline in mean accuracy (from 0.86 to 0.83); see Figure S2A. This result indicates that in complex, higher-order junctions, coaxial stacking cannot be fully captured by pairwise stem features alone and that topological context plays a non-negligible role. Junction-Explorer partially addressed this through features involving interactions between a stem pair and neighboring stems [49]. While such interaction energies may influence whether two stems preferentially stack, we deliberately limited the use of global descriptors in gCoSRNA. This decision reflects two considerations: first, high-order junction data are relatively sparse, which may compromise the robustness of global features, and second, the reconstruction step in gCoSRNA implicitly accounts for contributions from neighboring stems.

## 4. Discussion

The design of gCoSRNA is grounded in the principle of structural modularity [49], wherein complex multibranch junctions are decomposed into pseudo two-way junctions units. This strategy offers two main advantages. First, it eliminates the need to train separate models for different junction orders, allowing a single unified classifier to be applied across diverse topologies. Second, it effectively enlarges the training dataset by extracting multiple pseudo two-way units from higher-order junctions, thereby alleviating the common problem of data scarcity in multi-way junction modeling. Together, these features contribute to the model’s strong robustness and generalizability across a wide range of RNA structures. Importantly, our results show that the pairwise decomposition framework consistently delivers strong predictions across junctions of varying orders, implying that stem–stem stacking signatures captured at the local level may extend beyond specific junction contexts.

**Figure 3:**
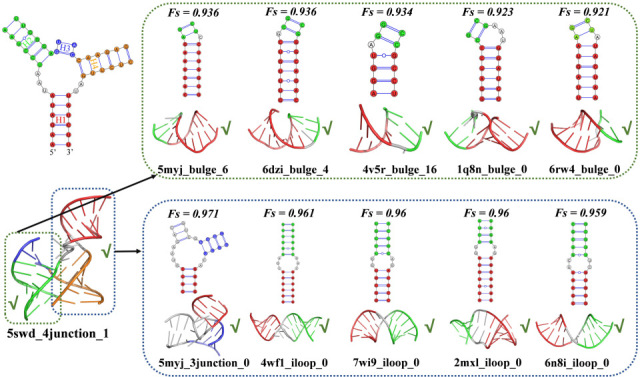
The positive impact of the modular decomposition strategy used in gCoSRNA. The 2D (**top**) and 3D (**bottom**) structures of the top five training set junctions most similar (based on feature vectors) to the H1–H4 (**right**, **bottom**) and H2–H3 (**right**, **top**) pseudo two-way junctions derived from the four-way junction 5swd_4junction_1 (**left**). In all 2D and 3D visualizations, stem elements are color-coded consistently to highlight structural correspondence (H1: red; H2: green; H3: blue; H4: orange; loops or regions unrelated to the current junction: grey), and √ indicates coaxial stacking between the two stems in a pseudo two-way junction.

To further assess the validity of this decomposition–reconstruction strategy, we conducted a case study based on feature similarity analysis. Specifically, we calculated a cosine similarity score (Fs) between the 12-dimensional feature vectors of pseudo two-way junctions in the test set and all samples in the training set. As illustrated in Figure 3, we examined a representative example from the test set, a four-way junction (5swd_4junction_1) containing two native coaxial stacking interactions: H1–H4 (red/orange) and H2–H3 (green/blue). We extracted two pseudo two-way motifs (H1–H2 and H3–H4) and compared each to the training set. The five most similar samples for each pseudo motif were visualized in 2D and 3D. Strikingly, all top matches originated from real two-way junctions (i.e., hairpins with internal loops or bulges) or three-way junctions rather than from other four-way junctions. This finding demonstrates that key stacking features are shared between pseudo junctions derived from complex multibranch loops and native two-way junctions. It highlights that gCoSRNA effectively learns generalizable stacking principles that transfer across different topologies.

**Figure 4:**
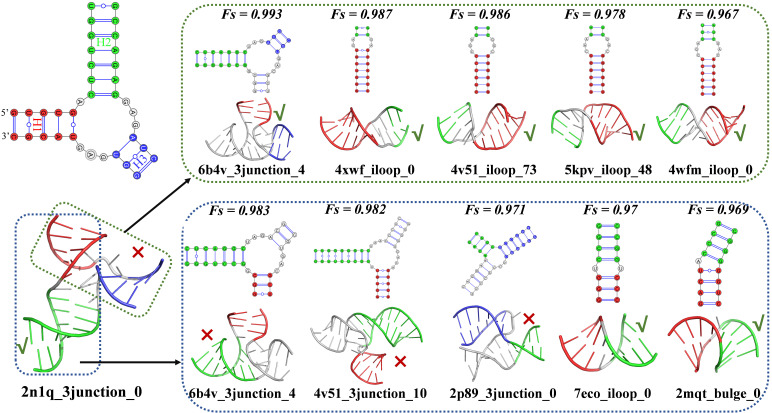
The limitations of the modular decomposition strategy in gCoSRNA. The 2D and 3D structures of the top five training set junctions most similar to the H1–H2 (bottom, red and green) and H1–H3 (top, red and blue) pseudo two-way junctions derived from the three-way junction 2n1q_3junction_0. All stem elements are consistently colored across 2D and 3D representations. In a pseudo two-way junction, √ and × denote coaxial stacking and non-stacking between the two stems, respectively.

Despite the overall effectiveness of the modular decomposition strategy, certain failure cases reveal its limitations. As shown in Figure 4, the RNA 2n1q_3junction_0 contains a three-way junction in which H1–H2 forms a coaxial stacking interaction in the native structure, yet gCoSRNA incorrectly predicts stacking between H1 and H3. Feature similarity analysis helps shed light on this discrepancy. For the pseudo two-way junction H1–H3, all of the top five most similar training instances (four from canonical two-way junctions and one from a three-way junction) exhibit coaxial stacking, potentially biasing the prediction. In contrast, for H1–H2, only two of the top five similar structures (both derived from two-way junctions) are coaxially stacked, while the remaining three (from three-way junctions) are not. This suggests that the model’s learned decision boundaries may be skewed when the pseudo two-way motifs originate from topologically distinct contexts, highlighting a trade-off between generalizability and structural specificity in the decomposition-based prediction framework.

**Figure 5:**
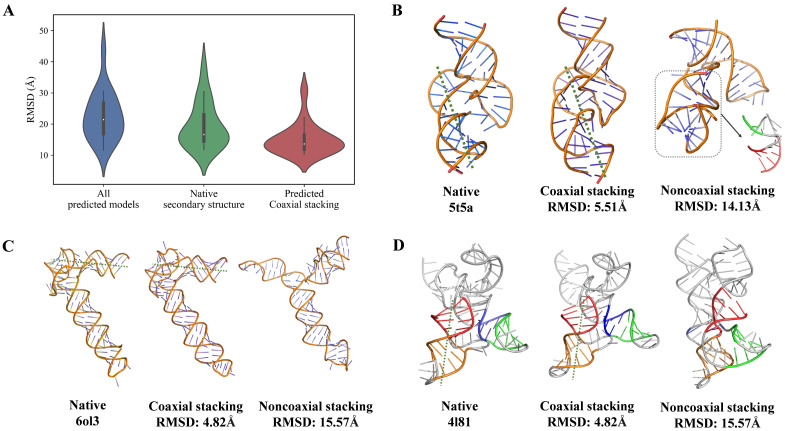
Coaxial stacking prediction as an indicator of 3D model accuracy. (**A**) The RMSD distributions of predicted RNA 3D structures for three- and four-way junctions in Test Set II, which includes CASP15/16 and RNA-Puzzles targets. From left to right: all submitted models (blue), models with high secondary structure fidelity (F1-score >0.8, green), and models that additionally match the coaxial stacking configuration predicted by gCoSRNA (red). (**B**–**D**). Representative examples of 3 three-/four-way junctions, each showing three structures (from left to right): the native structure, a predicted model with coaxial stacking consistent with gCoSRNA predictions, and a model with inconsistent stacking.

Given that coaxial stacking plays a critical role in shaping the 3D topology of RNA junctions, the accurate identification of stacking interactions is expected to enhance structural modeling accuracy [45,49]. To further evaluate the contribution of coaxial stacking prediction in RNA structure predictions, we conducted a comprehensive analysis on three- and four-way junctions from Test Set II, which includes structure prediction models submitted to CASP15/16 and RNA-Puzzles. As shown in Figure 5A (left), the overall distribution of RMSD values between predicted and native structures spans a wide range (~4–50 Å), with a mean of ~27.0 Å, reflecting large variability in structural accuracy. To control for differences in secondary structure, we first extracted base-pairing patterns using DSSR (version 1.8.1) and selected only models with a secondary structure F1-score > 0.8. These models displayed a more compact RMSD distribution (Figure 5A, middle), with a reduced mean RMSD of ~17 Å, representing a 59% improvement over the full set. From this secondary structure-consistent subset, we further identified models that correctly reproduced the coaxial stacking configurations predicted by gCoSRNA. This final group exhibited the most concentrated RMSD distribution (Figure 5A, right), with a mean RMSD of ~10 Å and that of the majority of models falling below 15 Å, yielding a ~70% improvement relative to all models. As illustrated in Figure 5B–D, for three representative multi-way junctions, incorporating coaxial stacking information led to a substantial improvement in structural accuracy, reducing the RMSD from over 14 Å to approximately 5 Å. It is worth noting, however, that the RNAs used for benchmarking often contain additional structural elements such as hairpins or single-stranded regions beyond the junction itself. Moreover, the global topology of a multi-way junction may be influenced not only by the stems involved in coaxial stacking but also by the angular relationships between them and other flanking stems within the junction (see Figure S16). As a result, the incorporation of stacking information does not always lead to a comparable reduction in the RMSD across all RNA targets, as observed in the representative examples. Nevertheless, these results underscore the importance of coaxial stacking as a higher-order structural determinant and demonstrate that gCoSRNA’s stacking predictions can serve as a reliable criterion for selecting high-quality 3D models from structure prediction pipelines.

Looking ahead, gCoSRNA can be integrated into RNA 3D structure prediction frameworks either as explicit energetic constraints or as post hoc evaluation criteria. Our previous coarse-grained modeling studies [21,23,64] demonstrated that including coaxial stacking interactions improves both structural accuracy and thermodynamic stability, particularly in two-way junctions [24,65,66]. Building on this foundation, a natural extension is to couple gCoSRNA with our coarse-grained simulations: junction conformations sampled during folding can be assessed by gCoSRNA, and the predicted stacking probabilities of pseudo two-way junctions can be translated into energetic constraints. Such a hybrid strategy would refine coaxial stacking parameters in a data-driven manner and improve the modeling of structurally complex junctions.

Despite its strong and generalizable performance, several limitations of gCoSRNA should be noted. First, although structurally diverse two-way junctions, such as hairpins containing bulges or internal loops, contribute to improved generalization, coaxial stacking cases in the PDB outnumber non-stacking instances by approximately sixfold (Figure S6). This substantial class imbalance may predispose the model to favor stacking predictions (Figure 4). Second, due to the limited availability of non-redundant RNA 3D structures, gCoSRNA (similar to Junction-Explorer [49]) relies on manually crafted features derived from the secondary structure. While such features offer interpretability (e.g., coaxial stacking free energy and connected loop length between two stems are identified as key contributors; see Figure S14), they may constrain predictive power and scalability. With future increases in available data, adopting deep learning strategies for automated feature extraction may enhance prediction accuracy and enable sequence-level coaxial stacking inference [37,40,67,68,69,70]. Third, unlike Junction-Explorer, which can also predict the global family type of an *n*-way junction [49,56], a property valuable for 3D modeling, gCoSRNA is currently trained on the pseudo two-way junctions formed by adjacent stems. As a result, it cannot directly infer junction family types or model coaxial stacking between non-adjacent stems in higher-order junctions, which remains an important direction for future extension. Fourth, gCoSRNA could be applied to predict coaxial stacking for multiple sequence variants and for cases occurring in exterior loops at RNA termini, but systematic evaluation remains challenging due to the scarcity of experimentally determined RNA 3D structures. In particular, limited structural data restrict the model’s ability to resolve subtle mutation-induced effects in junction regions, while exterior loop stacking may additionally be influenced by dangling ends and exhibits stability characteristics distinct from junction-derived stacking [71,72,73], making systematic evaluation difficult given the limited number of available structures. Fifth, although temperature and ionic conditions are critical determinants of RNA folding [21,45,65,66,73], gCoSRNA cannot currently account for them because most PDB training structures from X-ray crystallography or cryo-EM lack such metadata. Finally, although benchmarking on CASP and RNA-Puzzles targets shows that gCoSRNA helps identify structurally accurate models, the optimal strategy for integrating coaxial stacking predictions into existing RNA folding frameworks, particularly physics-based or deep learning-driven methods, remains an open question [9,74,75,76,77]. Addressing these challenges will be essential to further advancing RNA tertiary structure prediction and functional inference.

## 5. Conclusions

In this study, we developed gCoSRNA, a generalizable and topology-independent framework for predicting coaxial helical stacking in RNA multi-way junctions using only secondary structure-derived features. By decomposing complex junctions into pseudo two-way stem pairs and applying a unified machine learning model, gCoSRNA captures shared stacking signatures across diverse junction types and achieves accurate predictions without relying on junction-specific classifications. Systematic evaluations on three- to seven-way junctions demonstrate strong predictive performance and robust generalization. When integrated into 3D modeling workflows, gCoSRNA’s stacking predictions substantially improve structural fidelity, highlighting the biological relevance of coaxial stacking as a key determinant of RNA tertiary topology.

## Figures and Tables

**Table 1:** Prediction accuracy of different methods on two benchmark test sets.

Test Sets ^a^		Size	Junction-Explorer	Junction-Explorer-n	gCoSRNA
Test Set I	Two-way	144	- ^b^	-	0.90
	Three-way	15	0.73	0.73	0.80
	Four-way	17	0.25	0.76	0.76
	Five-way	3	-	-	0.93
	Six-way	1	-	-	0.83
	Seven-way	3	-	-	1.0
Test Set II	Two-way	77	-	-	0.95
	Three-way	9	0.55	0.66	0.66
	Four-way	28	0.53	0.68	0.75

^a^ For two- to four-way junctions, the reported values represent full-configuration prediction accuracy. For junctions beyond the five-way level, pairwise accuracy (PA) is used. The PA values shown represent the average PA across all structures in the corresponding dataset. ^b^ “-” indicates that the prediction was not performed due to the absence of a corresponding trained model/parameters for the specified junction type in the original RNA Junction-Explorer implementation.

## Data Availability

The training set, test set, and source code are freely available at https://github.com/RNA-folding-lab/gCoSRNA (accessed on 30 September 2025).

## Supplementary Materials

The following supporting information can be downloaded at https://www.mdpi.com/article/10.3390/biom16020230/s1, Figure S1: Schematic illustration of a three-way junction decomposed into three pseudo two-way junctions with annotated structural features; Figure S2: Contribution and illustration of the topological descriptor *N*; Figure S3: Workflow for constructing a non-redundant RNA junction dataset; Figure S4: Characteristics of RNA-only structures and junctions in the dataset used in this work; Figure S5: Schematic illustration of decomposing a complex RNA secondary structure into individual junctions; Figure S6: Junction composition and coaxial stacking patterns in the two test sets; Figure S7. Probability distribution of coaxial stacking predictions for pseudo two-way junctions in the training set; Figure S8: Representative examples of possible coaxial stacking configurations in a three-way RNA junction; Figure S9. Illustration of pairwise accuracy (PA) calculation using a five-way RNA junction; Figure S10: Performance of gCoSRNA evaluated via 10-fold cross-validation on the pseudo two-way junction training set; Figure S11: Representative examples of five-, six-, and seven-way RNA junctions in Test Set I; Figure S12: Examples of mispredicted coaxial stacking cases in Test Set II, likely influenced by ligand or ion binding; Figure S13: Comparison of gCoSRNA with the physics-based method of Tyagi and Mathews; Figure S14: Feature importance analysis for the gCoSRNA model; Figure S15: Feature importance analysis of individual stem properties; Figure S16: Structural comparison for RNA target 4qlm; Table S1: Performance metrics (accuracy, precision, and F1-score) for all pseudo two-way junctions derived from multi-branch junctions of each order in the test set, treating them as a whole in the binary classification task; Table S2: Comparisons of the prediction accuracy between the gCoSRNA model and the physics-based method of Tyagi and Mathews across their benchmark datasets for three- and four-way junctions; Text S1: Evaluation Metrics.
