# Supplementary material for: gCoSRNA: Generalizable Coaxial Stacking Prediction for RNA Junctions Using Secondary Structure
Source: Biomolecules. 2026 Feb 2;16(2):230. doi: 10.3390/biom16020230 (PMC12938077; doi:10.3390/biom16020230)
Supplement: Supplementary file 1 [file biomolecules-16-00230-s001.zip › biomolecules-4027068-supplementary-revised.pdf]

Supplementary Materials for

gCoSRNA: Generalizable Coaxial-Stacking Prediction for RNA Junctions Using Secondary Structure

Shasha Li[1], Qianqian Xu[1], Ya-Lan Tan[2], Jian Jiang[1], Bengong Zhang[1], Ya-Zhou Shi[*,1]

[1] Center for Applied Mathematics and Interdisciplinary Sciences, School of Mathematics & Statistics, Wuhan Textile University, Wuhan 430200, China.

[2] School of Bioengineering and Health, Wuhan Textile University, Wuhan 430200, China.

* Correspondence: yzshi@wtu.edu.cn (YS)

Text S1: Evaluation Metrics

To evaluate the performance of the proposed classification model, we used standard metrics including accuracy, *F1*-score, macro-averaged *F1*-score, Cohen's Kappa coefficient, the confusion matrix, the receiver operating characteristic (ROC) curve, and the area under the ROC curve (AUC).

The confusion matrix summarizes model predictions in terms of true positives (TP), true negatives (TN), false positives (FP), and false negatives (FN). Based on these values, key evaluation metrics are computed:

(1) Accuracy is defined as the proportion of correctly classified samples:

$$Accuracy = \frac{TP + TN}{TP + TN + FP + FN} \tag{S1}$$

(2) Precision and recall for the positive class are defined as:

$$Precision = \frac{TP}{TP + FP} \tag{$S2$}$$

$$Recall = \frac{TP}{TP + FN} \tag{S3}$$

(3) F1-score is the harmonic mean of precision and recall:

$$F1 = 2 * \frac{Precision * Recall}{Precision + Recal} \tag{$S4$}$$

(4) To assess classification agreement beyond chance, we also computed Cohen's Kappa coefficient (κ), which accounts for the possibility of random agreement. It is defined as:

$$k = \frac{p_o - p_e}{1 - p_e}, \tag{$S4$}$$

where p_o is the observed agreement (i.e., accuracy), and p_e is the expected agreement by chance:

$$p_e = \frac{(TP + FP)(TP + FN) + (FN + TN)(FP + TN)}{(TP + FP + FN + TN)^2}. \tag{$S5$}$$

Kappa values range from –1 to 1, where 1 indicates perfect agreement, 0 indicates chance-level agreement, and negative values suggest systematic disagreement.

(5) Finally, we used the ROC curve to evaluate the model's ability to distinguish between classes across thresholds. The AUC quantifies overall discriminative performance, with 1.0 indicating perfect separation and 0.5 representing random guessing.

(6) To quantitatively assess coaxial stacking prediction in multi-branch junctions, we introduce a new evaluation metric: pairwise accuracy (PA). In an n-way junction, there are N adjacent stem pairs, each forming a pseudo two-way substructure. For each pair, the model predicts whether coaxial stacking occurs. The *PA* score is defined as the proportion of these stem pairs whose stacking status is correctly predicted:

$$PA = \frac{1}{n}\sum_{(i,j)} 1[pred_{ij} = y_{ij}], \tag{$S6$}$$

where n is the number of cyclically adjacent stem pairs (H_i, H_{i+1}) in an n-way junction, y_{ij} denotes the ground truth (stacked / unstacked), and $pred_{ij}$ is the model prediction; see an example in Fig. S12.

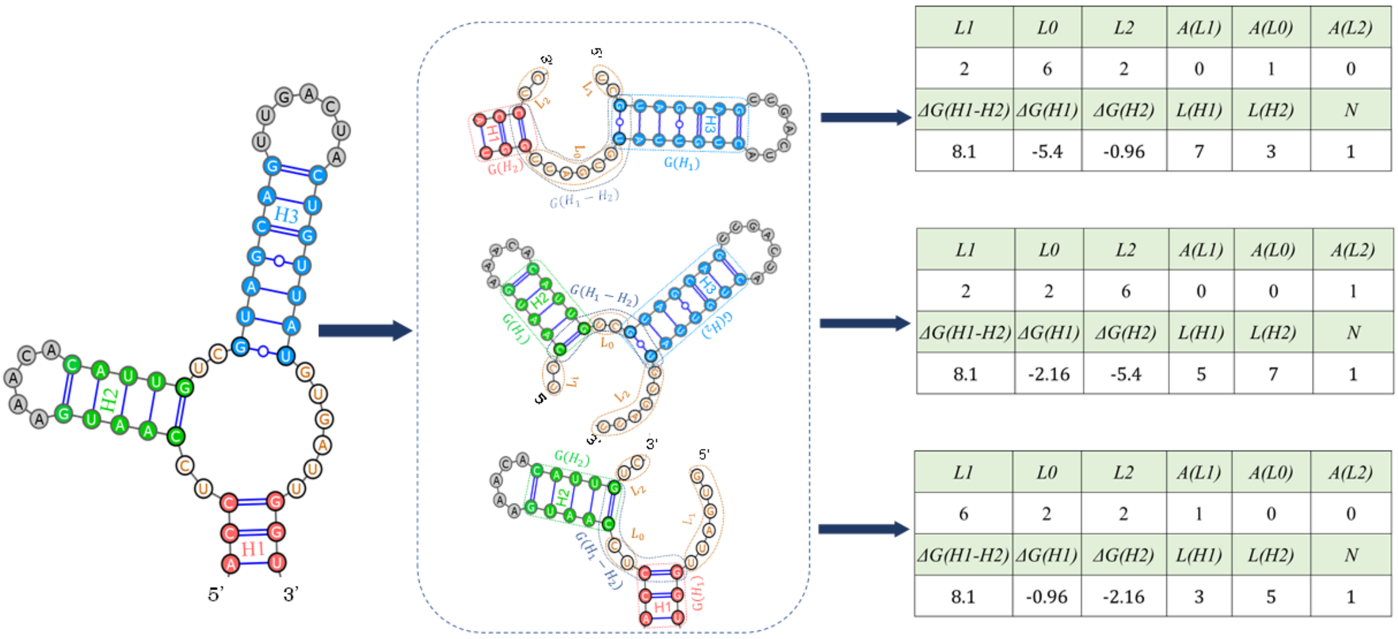

L1	L0	L2	A(L1)	A(L0)	A(L2)
2	6	2	0	1	0
ΔG(H1-H2)	ΔG(H1)	ΔG(H2)	L(H1)	L(H2)	N
8.1	-5.4	-0.96	7	3	1

L1	L0	L2	A(L1)	A(L0)	A(L2)
2	2	6	0	0	1
ΔG(H1-H2)	ΔG(H1)	ΔG(H2)	L(H1)	L(H2)	N
8.1	-2.16	-5.4	5	7	1

L1	L0	L2	A(L1)	A(L0)	A(L2)
6	2	2	1	0	0
ΔG(H1-H2)	ΔG(H1)	ΔG(H2)	L(H1)	L(H2)	N
8.1	-0.96	-2.16	3	5	1

Figure S1. Schematic illustration of a three-way junction decomposed into three pseudo two-way junctions with annotated structural features. Left: The original three-way junction consists of three helices: H1, H2, and H3. Middle: It is decomposed into three pseudo two-way substructures: H1–H2, H2–H3, and H3–H1, each comprising a pair of adjacent stems and the connecting loop region, and key structural features are annotated for each substructure. Right: Features values for each pseudo two-way junction.

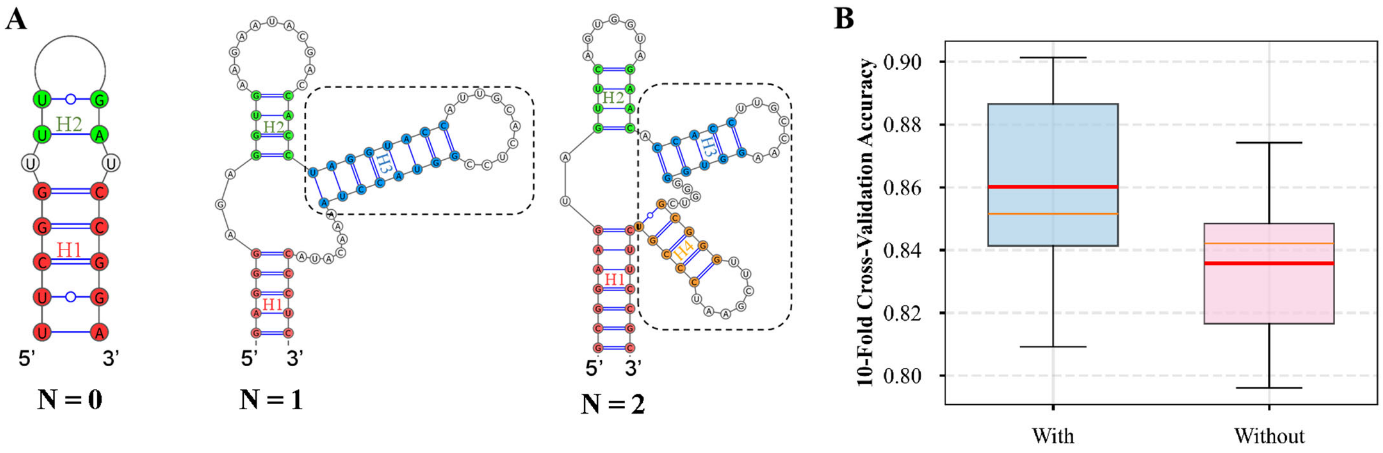

Figure S2. Contribution and illustration of the topological descriptor *N*. (A) Illustration of how *N* is defined for a stem pair H1–H2: for a two-way junction (e.g., a hairpin with an internal loop), $N = 0$ (no intervening stems); for a three-way and four-way junction, $N = 1$ and $N = 2$, respectively. (B) Comparison of ten-fold cross-validation accuracies of gCoSRNA models with and without inclusion of feature *N*.

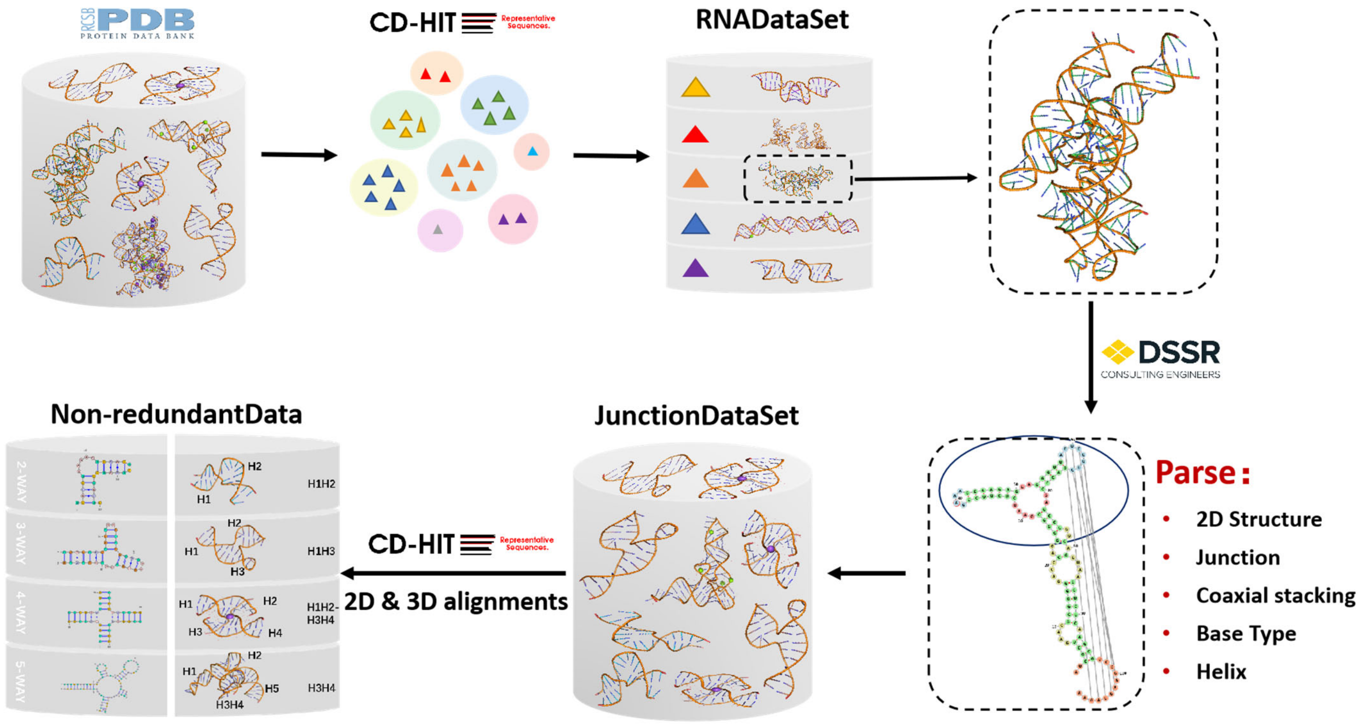

Figure S3. Workflow for constructing a non-redundant RNA junction dataset. RNA-only structures were first collected from the PDB and clustered using CD-HIT to generate a non-redundant set of representative RNA structures. Each structure was then parsed using DSSR to extract individual n-way junctions, yielding a comprehensive junction dataset. All these junctions were further clustered by CD-HIT, followed by secondary and tertiary structure alignments to remove redundancy at the junction level, resulting in the final non-redundant RNA junction dataset.

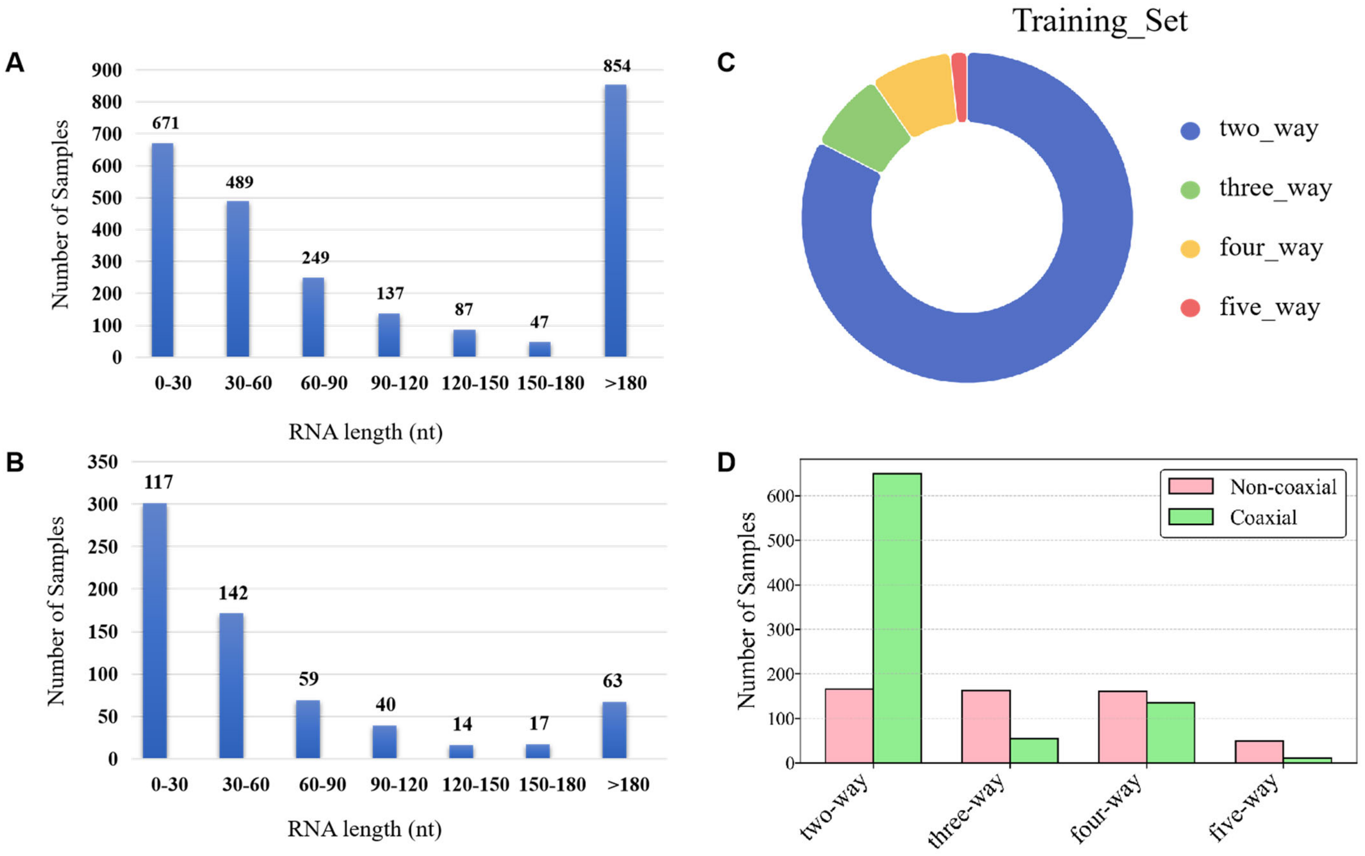

Figure S4. Characteristics of RNA-only structures and junctions in the dataset used in this work. A. Length distribution of all RNA-only structures derived from PDB. **B.** Length distribution of RNA sequences in the non-redundant dataset after CD-HIT clustering. **C.** Proportion of multibranch junctions with different numbers of helices (i.e., n-way junctions) in the training set. **D.** Distribution of coaxially stacked versus non-stacked adjacent pseudo-stem pairs across different types of n-way junctions in the training set.

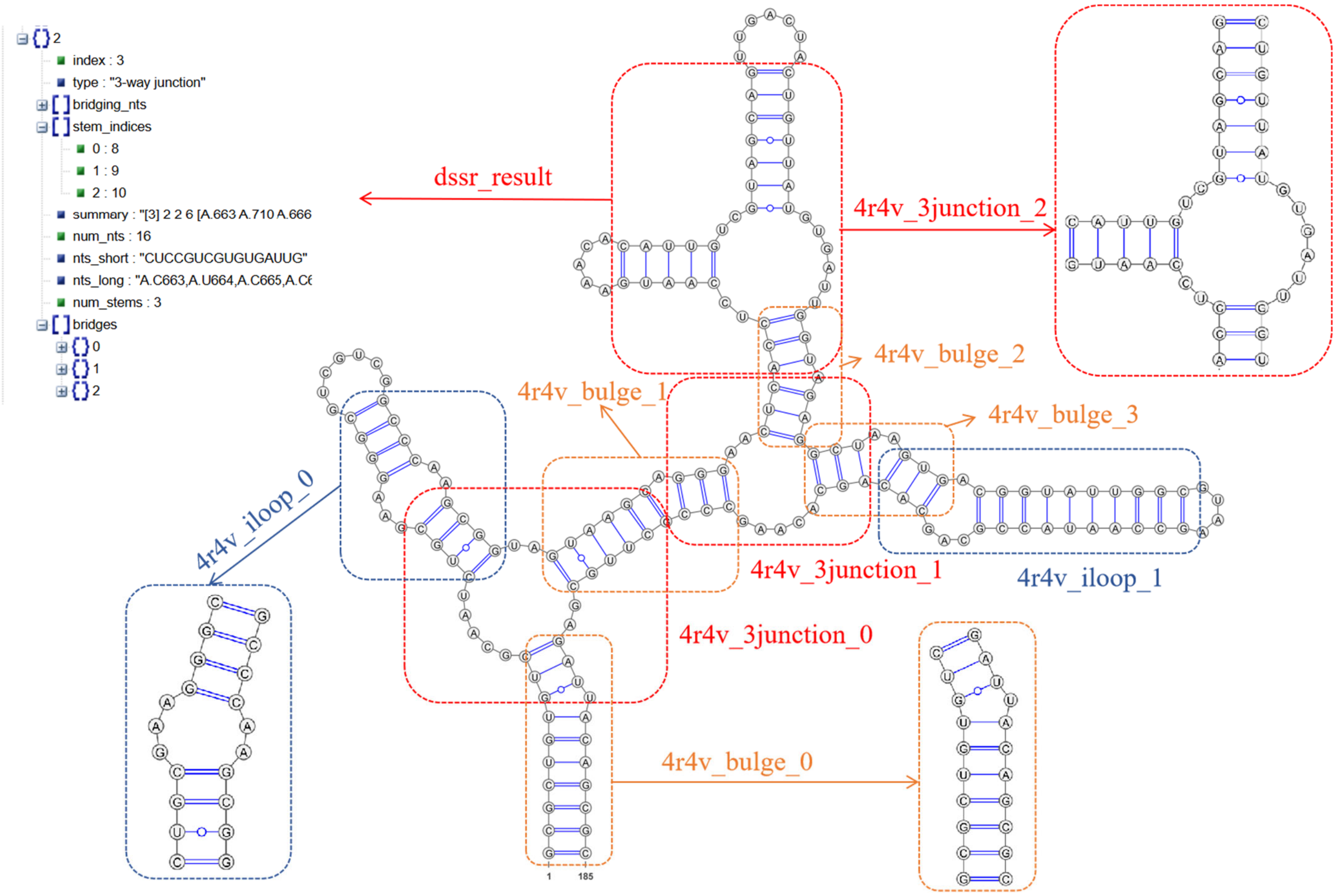

Figure S5. Schematic illustration of decomposing a complex RNA secondary structure into individual junctions. For a given PDB structure (e.g., PDB ID: 4r4v), DSSR is used to parse the RNA and identify all junctions, including their junction types, stem base-pairings, and loop sequences. Based on this information, each junction that defined as the junction loop together with all directly connected stems, is treated as an independent structural unit and renamed following a unified convention: PDB ID_junction type_index (e.g., 4r4v_bulge_0, or 4r4v_3junction_0), where the index denotes the i-th junction of the same type along the 5′-3′ direction within a given chain. The corresponding sequence, secondary structure, chain ID, and nucleotide number of each junction are then extracted and recorded (see at github: https://github.com/RNA-folding-lab/gCoSRNA/tree/main/Datasets).

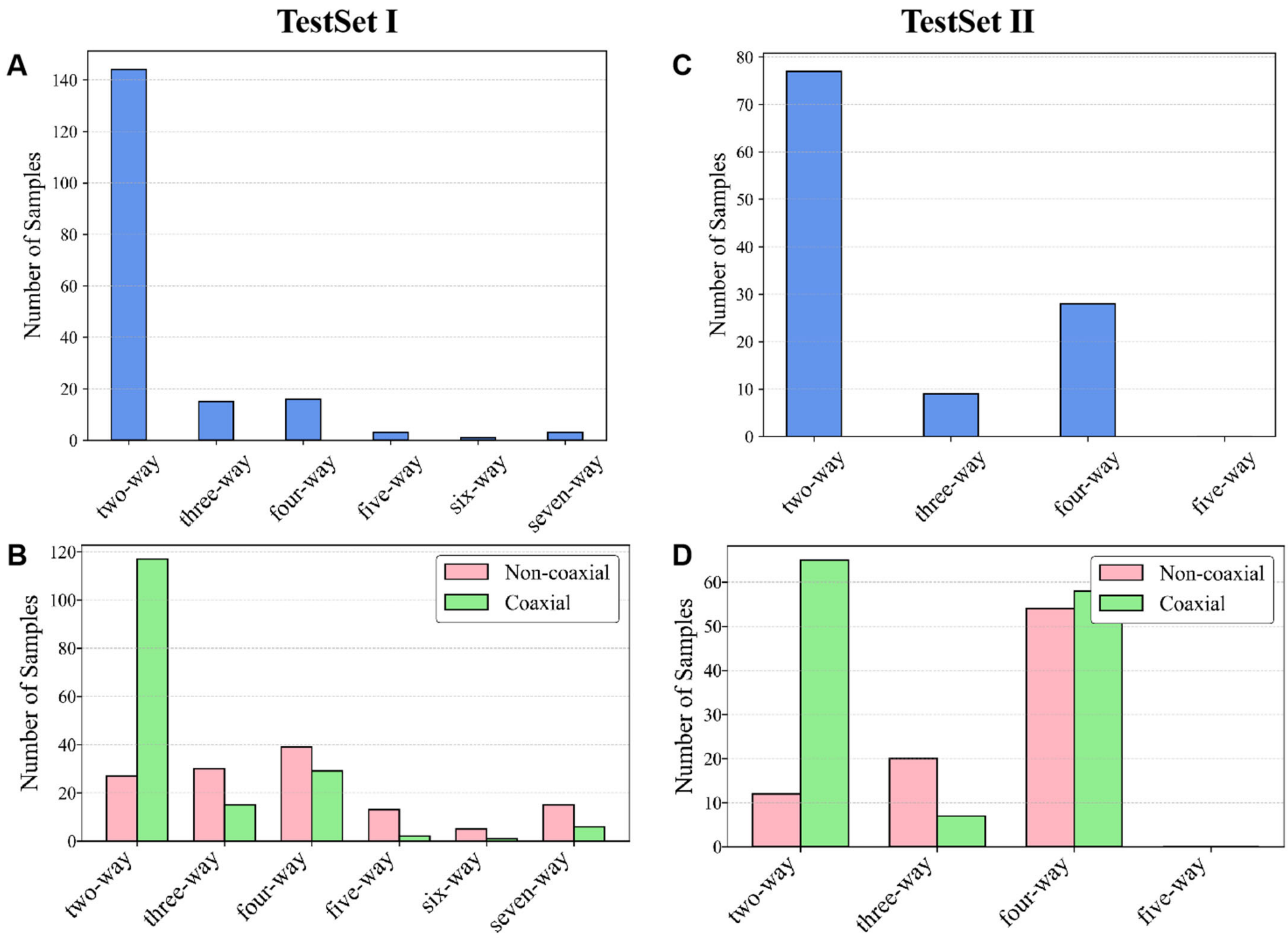

Figure S6. Junction composition and coaxial stacking patterns in the two test sets. A, C. Distribution of junctions with different branches in Test Set I (A) and II (C). **B, D.** The number of coaxially stacked versus non-stacked adjacent pseudo-stem pairs across different junction types in Test Set I (B) and II (D).

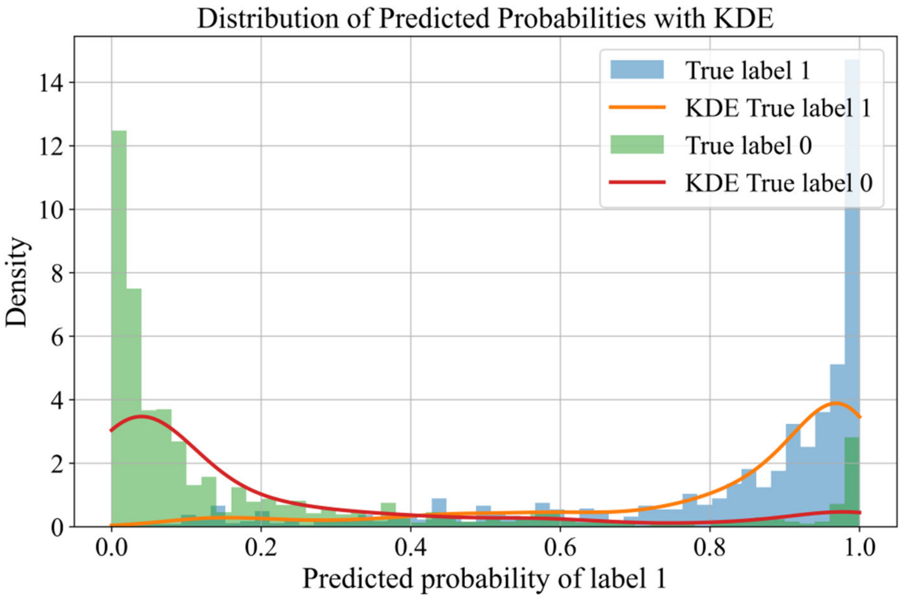

Figure S7. Probability distribution of coaxial stacking predictions for pseudo two-way junctions in the training set. The histogram and kernel density estimation (KDE) curves show the predicted coaxial stacking probabilities for positive (coaxially stacked) and negative (non-stacked) samples. The two distributions intersect at ~0.42, which was selected as the classification threshold for determining coaxial stacking.

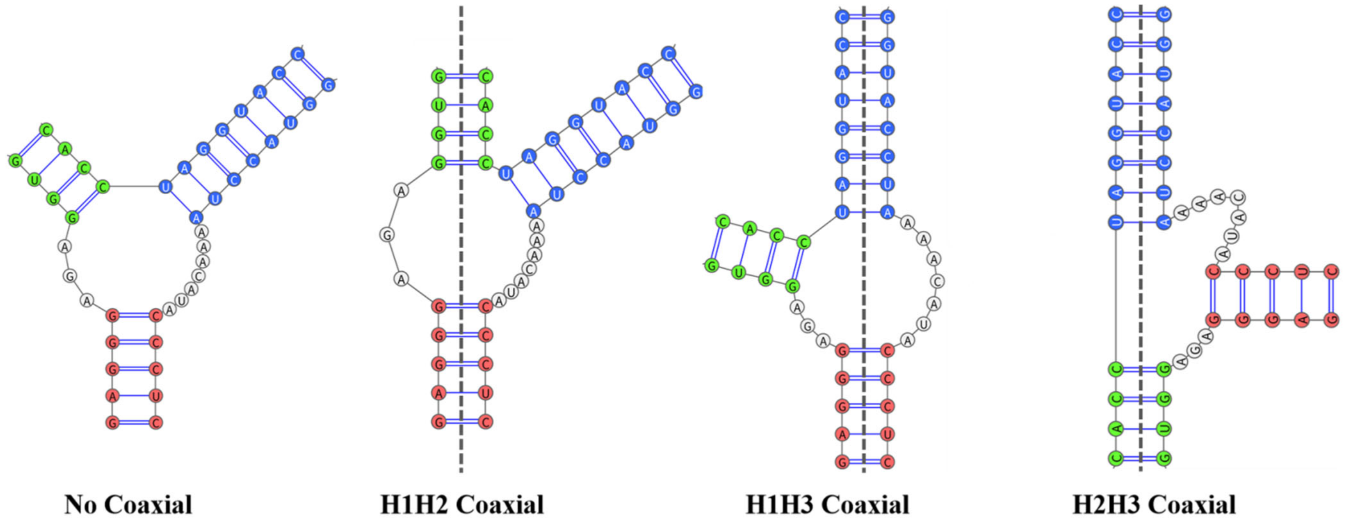

Figure S8. Representative examples of possible coaxial stacking configurations in a three-way RNA junction. From left to right: (1) no coaxial stacking, (2) coaxial stacking between H1 and H2, (3) between H1 and H3, and (4) between H2 and H3. Different helices are color-coded (H1: red; H2: green; H3: blue) for clarity.

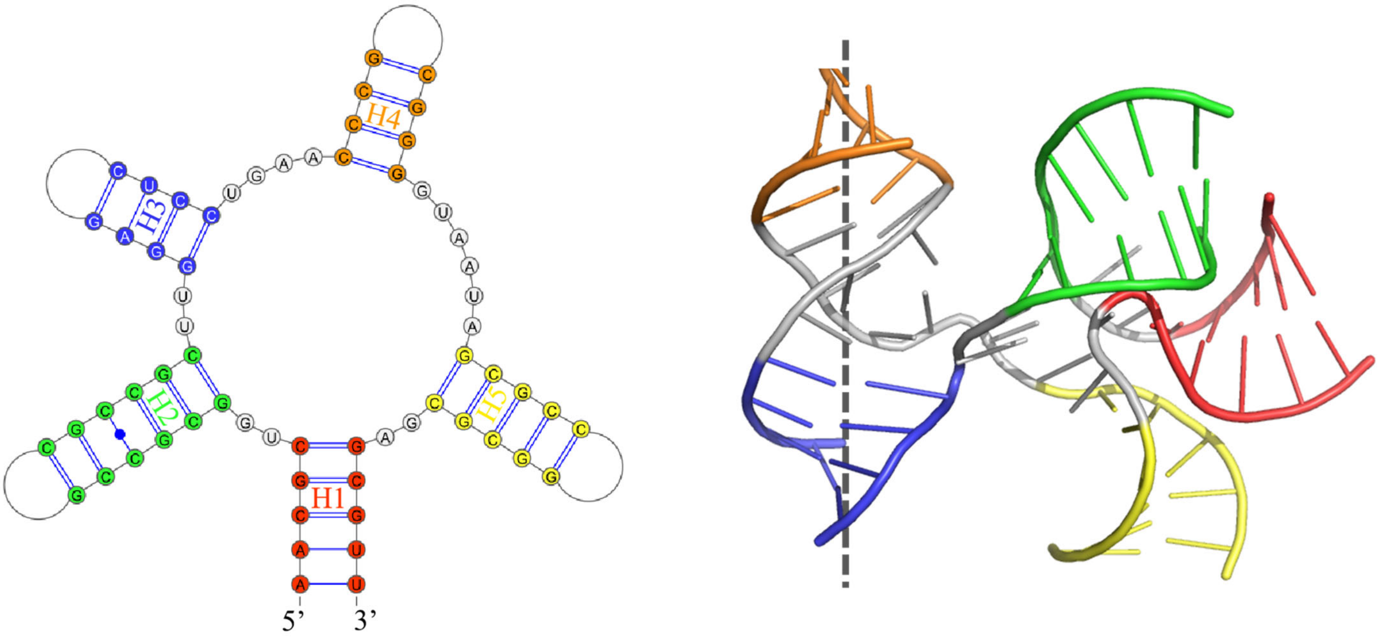

Figure S9. Illustration of pairwise accuracy (PA) calculation using a five-way RNA junction. The junction consists of five adjacent helix pairs: (H1, H2), (H2, H3), (H3, H4), (H4, H5), and (H5, H1). Among them, only (H3, H4) forms a coaxial stacking interaction in the native tertiary structure. The left panel shows the RNA secondary structure, while the right panel presents the corresponding tertiary structure. Each helix is color-coded for clarity. The ground-truth label vector for coaxial stacking is thus 0, 0, 1, 0, 0, and a prediction matching this exactly would yield a *PA* score of 1.0.

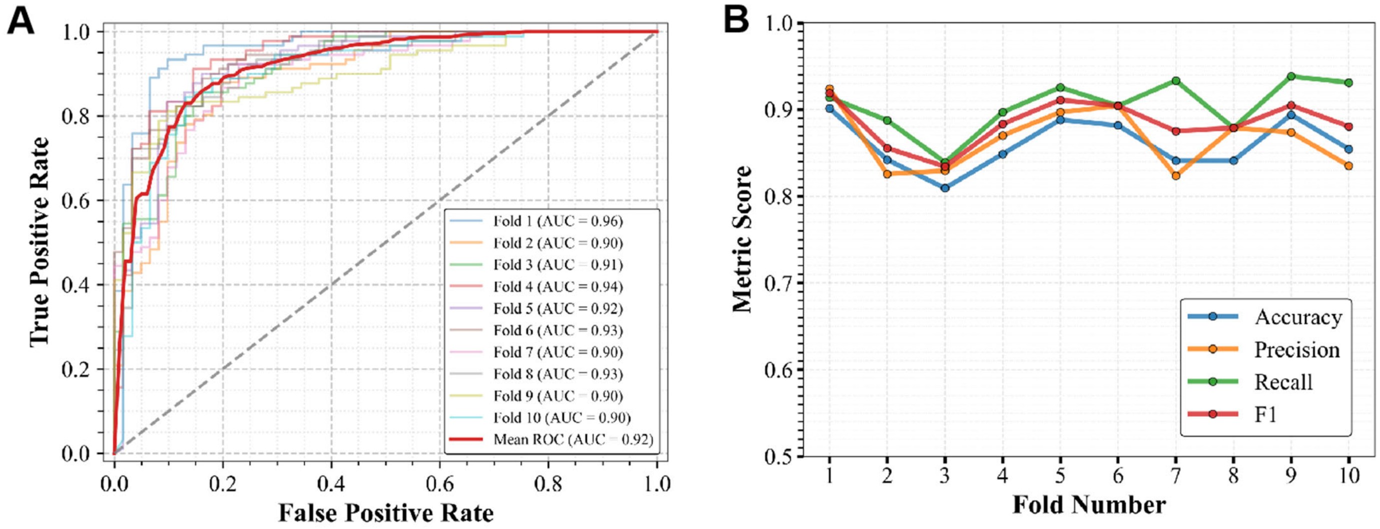

Figure S10. Performance of gCoSRNA evaluated via 10-fold cross-validation on the pseudo two-way junction training set. A. ROC curves for each individual fold (light lines) and the averaged ROC curve (bold line). **B.** Cross-validation performance metrics (i.e., accuracy, precision, recall, F1-score, and Cohen's Kappa) across the 10 folds.

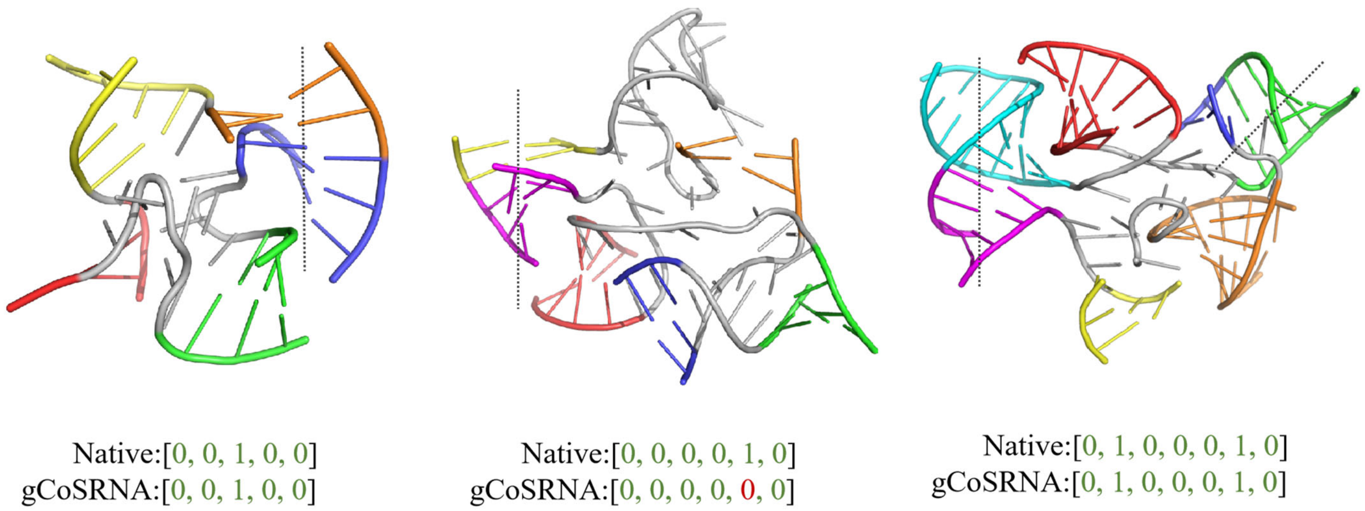

Figure S11. Representative examples of five-, six-, and seven-way RNA junctions in Test Set I. Each panel illustrates the 3D structure of a multibranch junction (five-way, six-way, or seven-way), with individual helices shown in distinct colors. Native coaxial stacking interactions are indicated and compared with gCoSRNA prediction results.

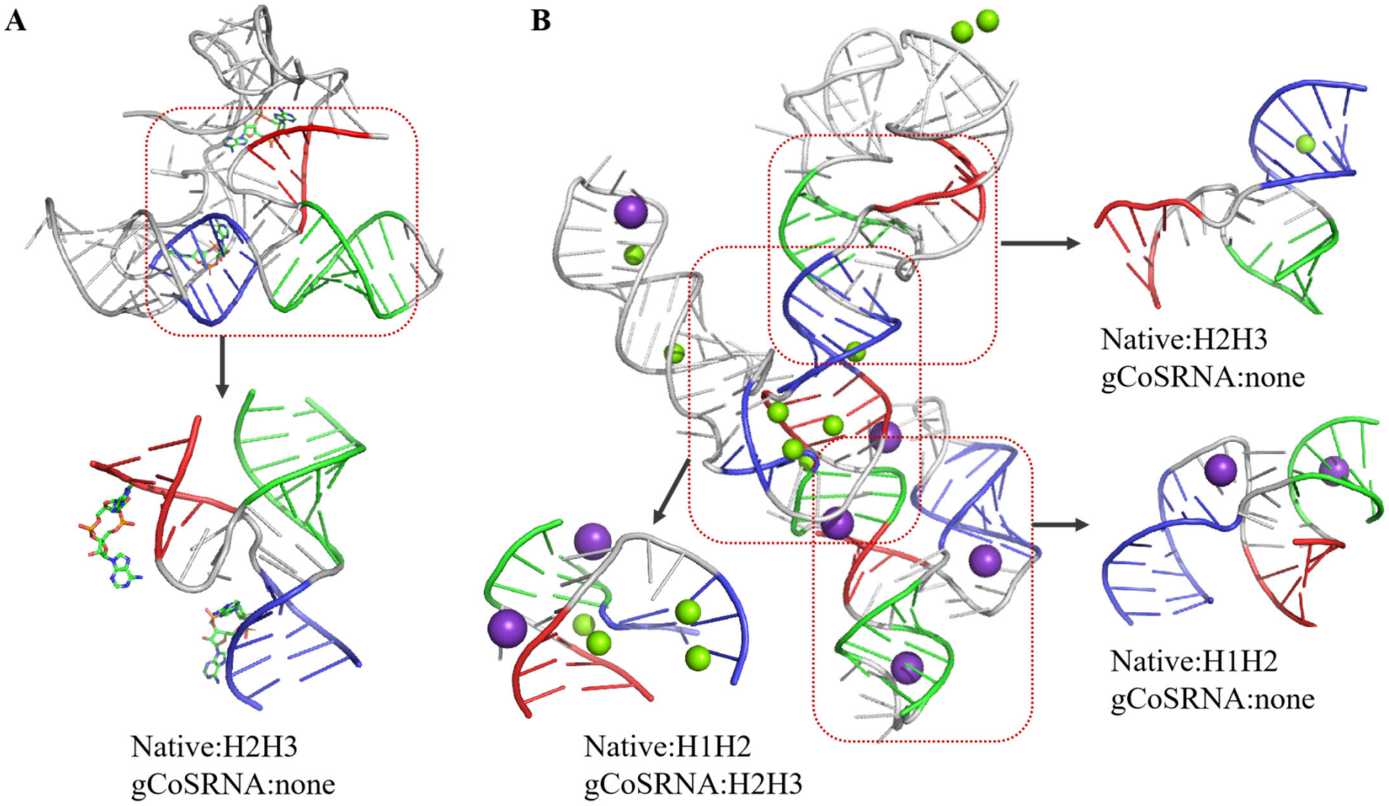

Figure S12. Examples of mispredicted coaxial stacking cases in Test Set II, likely influenced by ligand or ion binding. All structures shown are experimentally solved PDB structures. Dotted lines circle the junction regions extracted from the full RNA structures. **A.** RNA 4QLM contains a single three-way junction. Although gCoSRNA predicted no coaxial stacking in this junction, the native tertiary structure shows stacking between helices H2 and H3 (green and blue), which is likely induced by interactions with two ligands. **B.** RNA 4R4V includes three distinct three-way junctions: 4r4v_3junvtion_0, 4r4v_3junvtion_1, and 4r4v_3junvtion_2. For 4r4v_0, gCoSRNA predicted H2–H3 stacking due to the absence of unpaired nucleotides between them and longer loop regions elsewhere. However, stacking is observed between H1 and H2 (red and green) in the native structure, potentially induced by bound potassium and magnesium ions. The stacking configurations of 4r4v_3junvtion_1 and 4r4v_3junvtion_2 resemble that of 4QLM, and both were also mispredicted by the model. For each junction, the native coaxial stacking in the extracted substructure and the corresponding gCoSRNA prediction are indicated. These cases highlight the challenges in predicting stacking when external molecular factors, such as ligands or ions, alter local RNA geometry.

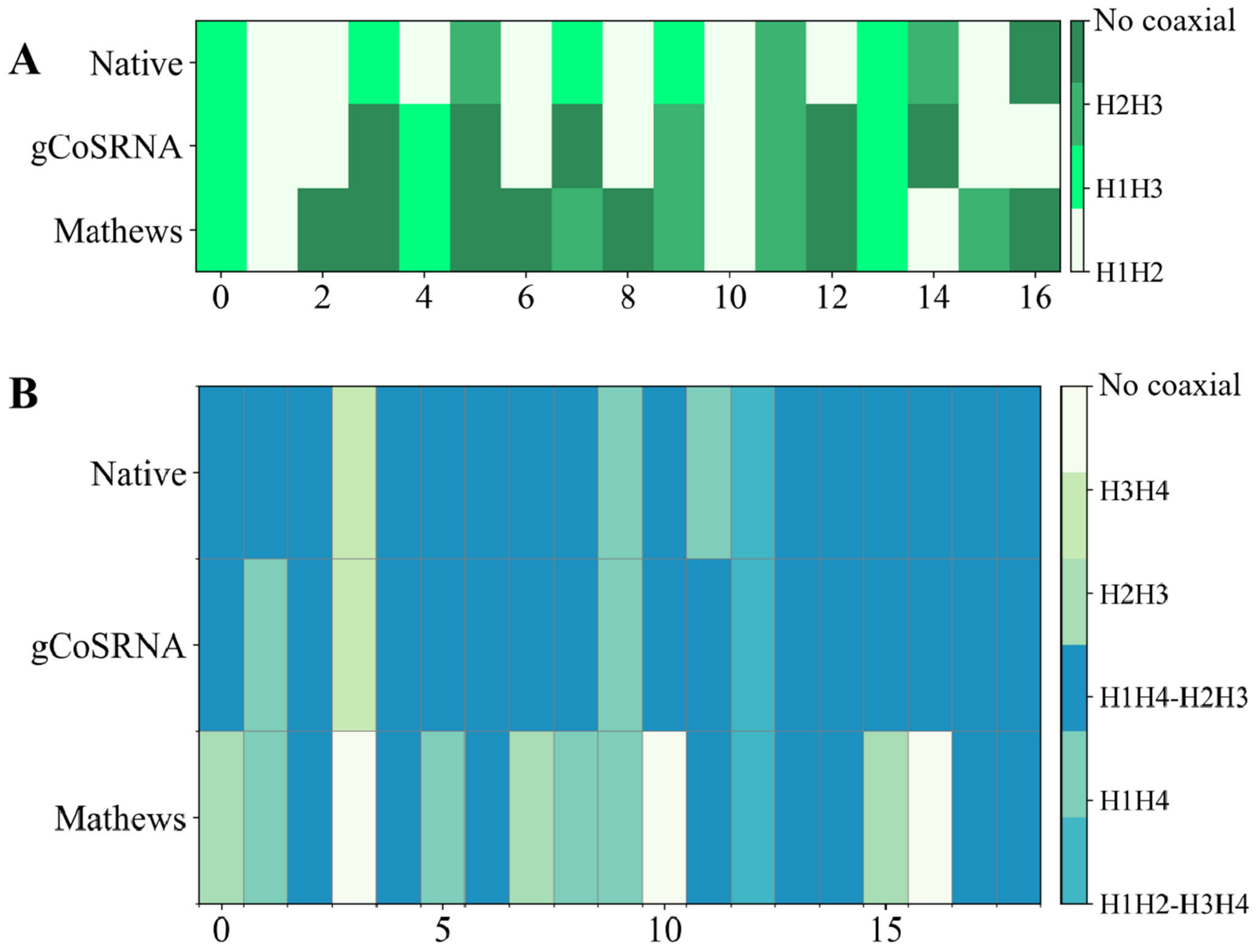

Figure S13. Comparison of gCoSRNA with the physics-based method of Tyagi and Mathews. (A) Three-way junctions. (B) Four-way junctions. Each panel shows the native configuration (top) and the predictions by gCoSRNA (middle) and Tyagi and Mathews (2007) (bottom).

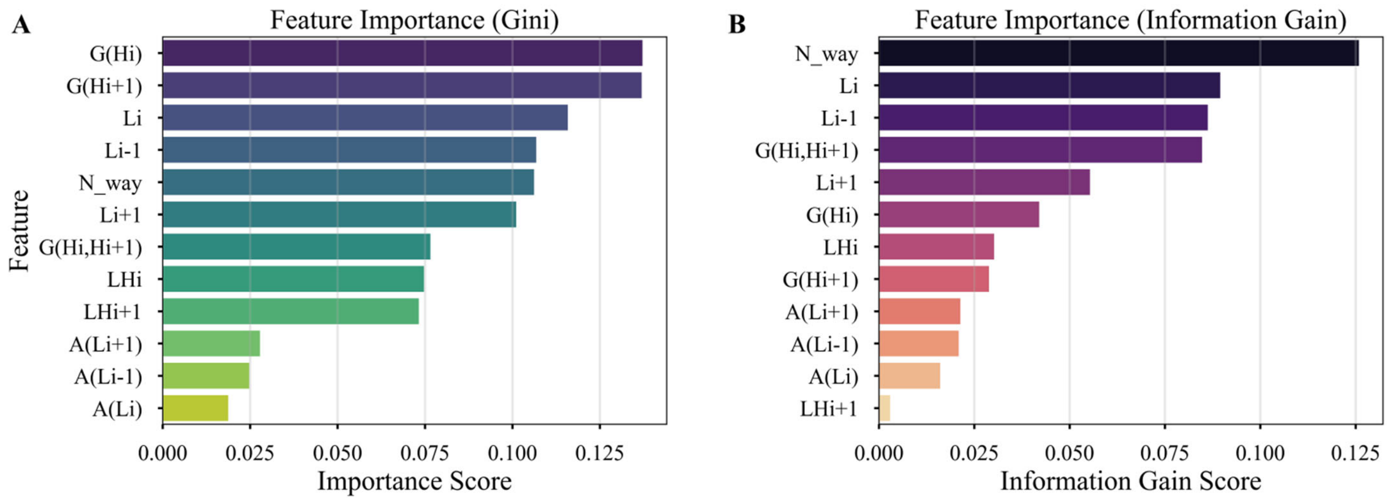

Figure S14. Feature importance analysis for the gCoSRNA model. A. Feature importance ranked by Gini impurity (used in the random forest algorithm), reflecting how each feature contributes to reducing classification error through node splitting. **B.** Feature importance ranked by information gain, quantifying how much uncertainty each feature reduces during the training process.

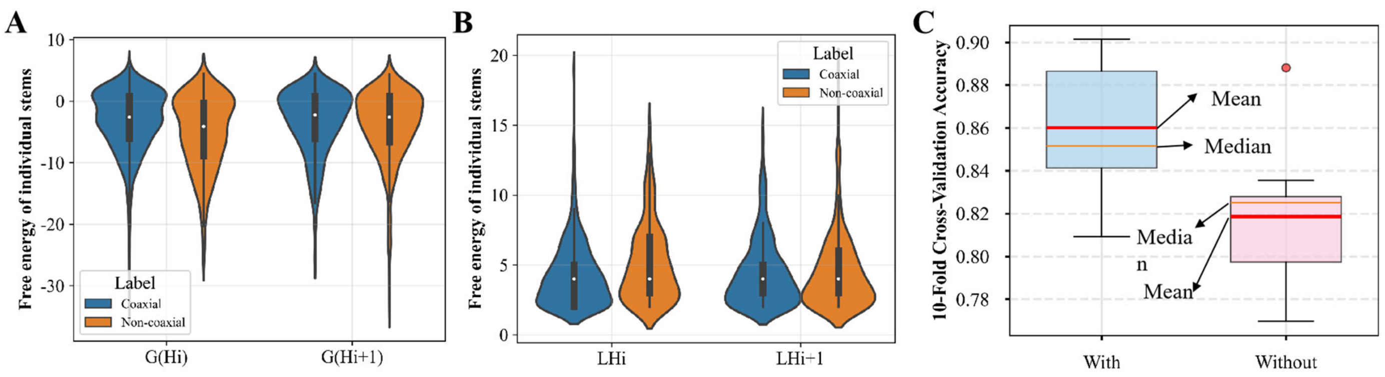

Figure S15. Feature importance analysis of individual stem properties. (A, B) Distributions of free energy (A) and stem length (B) for coaxial-stacked versus non-coaxial-stacked pseudo two-way junctions in the training dataset. (C) Comparison of ten-fold cross-validation accuracies of gCoSRNA models with and without inclusion of stem length and free energy as features.

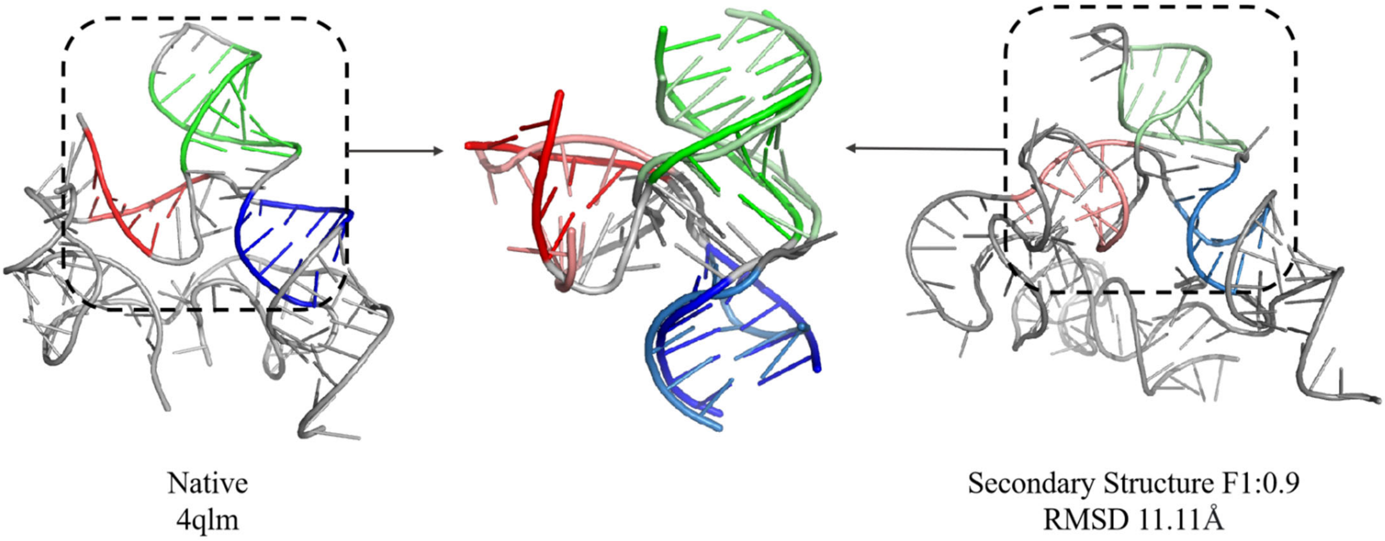

Figure S16. Structural comparison for RNA target 4qlm. The left and right panels show the native and predicted structures, respectively. Although the predicted model has a global RMSD of ~11 Å, the superposition of the 3-way junction fragments (middle) demonstrates that the local coaxial stacking topology was correctly reconstructed, consistent with gCoSRNA predictions.

Table S1. Performance metrics (accuracy, precision, and F1-score) for all pseudo two-way junctions derived from multi-branch junctions of each order in the test set, treating them as a whole in the binary classification task.

Test Sets		Number	Accuracy	Precision	F1-score
Testset Ⅰ	Two-way	144	0.90	0.90	0.94
	Three-way	15	0.89	0.91	0.81
	Four-way	17	0.71	0.76	0.77
	Five-way	3	0.93	1	0.66
	Six-way	1	0.83	0	0
	Seven-way	3	1	1	1
Testset Ⅱ	Two-way	77	0.95	0.97	0.97
	Three-way	9	0.85	0.8	0.66
	Four-way	28	0.81	0.88	0.8

Table S2. Comparisons of the prediction accuracy between the gCoSRNA model and the physics-based method of Tyagi and Mathews across their benchmark datasets for three- and four-way junctions.

	Number	Mathews	gCoSRNA
Three-way	17	0.35	0.53
Four-way	19	0.47	0.89
